# Short-Term and Imminent Rainfall Prediction Model Based on ConvLSTM and SmaAT-UNet

**DOI:** 10.3390/s24113576

**Published:** 2024-06-01

**Authors:** Yuanyuan Liao, Shouqian Lu, Gang Yin

**Affiliations:** 1 School of Computer Science and Technology, Xinjiang University, Urumqi 830049, China; 2 College of Geography and Remote Sensing Sciences, Xinjiang University, Urumqi 830049, China; chinayg@xju.edu.cn

**Keywords:** short-term precipitation forecasting, deep learning, radar image extrapolation, ConvLSTM, SmaAT-UNet

## Abstract

Short-term precipitation forecasting methods are mainly divided into statistical forecasting, numerical model-based forecasting, and radar image extrapolation techniques. The two methods based on statistical prediction and numerical model have the disadvantages of being unstable and generating large errors. Therefore, this study proposes the use of deep learning for radar image extrapolation for precipitation forecasting, in particular by developing algorithms for ConvLSTM and SmaAT-UNet. The ConvLSTM model is a fusion of a CNN (Convolutional Neural Network) and LSTM (Long Short-Term Memory network), which solves the challenge of processing spatial sequence data, which is a task that traditional LSTM models cannot accomplish. At the same time, SmaAT-UNet enhances the traditional UNet structure by incorporating the CBAM (Convolutional Block Attention Module) attention mechanism and replacing the standard convolutional layer with depthwise separable convolution. This innovative approach aims to improve the efficiency and accuracy of short-term precipitation forecasting by improving feature extraction and data processing techniques. Evaluation and analysis of experimental data show that both models exhibit good predictive ability, with the SmaAT-UNet model outperforming ConvLSTM in terms of accuracy. The results show that the performance indicators of precipitation prediction, especially detection probability (POD) and the Critical Success index (CSI), show a downward trend with the extension of the prediction time. This trend highlights the inherent challenges of maintaining predictive accuracy over longer periods of time and highlights the superior performance and resilience of the SmaAT-UNet model under these conditions. Compared with the statistical forecasting method and numerical model forecasting method, its accuracy in short-term rainfall forecasting is improved.

## 1. Introduction

Short-term precipitation is defined as rainfall events forecasted to occur within the ensuing few hours, typically constrained to a forecast horizon of up to 6 h. The precision of such forecasts can achieve remarkable granularity, extending to kilometer-level and minute-level accuracy [1]. The capacity to accurately predict short-term precipitation bears considerable significance across various sectors, including agriculture, transportation, and water management. By facilitating timely preemptive actions, it allows for effective mitigation of precipitation-related impacts.

With the advent of data mining and machine learning technologies, statistical forecasting methods have gained widespread applications [2]. Precipitation is a very complex nonlinear problem which involves the exchange of water, heat, and momentum between the ground and the air during the water cycle. However, statistical methods are criticized for their lack of a physical basis and inherent instability [3]. Given their linear nature, they struggle with the fundamentally nonlinear characteristics of meteorological elements, constraining forecast accuracy. This limitation has steered research towards nonlinear forecasting methods, prominently featuring artificial neural networks (ANNs). ANNs excel in mapping nonlinear relationships between input and output variables, reliant solely on training samples and targets. This capability allows for the selection of an optimal network configuration through learning and training processes, enhancing the model’s ability to generalize beyond the training data [4].

Numerical weather prediction is based on the input of radar, satellite, ground and upper air observations and topographic data, and the output of meteorological elements such as temperature and precipitation at a future time. Numerical weather precipitation forecasting offers several advantages but is not without drawbacks. Limitations such as the low spatial resolution of radar images, accumulative errors, and the influence of terrain and water bodies introduce significant uncertainty into forecast results. These factors adversely affect the practical application of this method’s accuracy and reliability. An innovative approach involves using radar extrapolation algorithms to predict future precipitation amounts and distribution based on radar-derived reflectivity factor data.

Noteworthy contributions to the field include the work by Sohail et al., who employed a BP neural network and an ARMA linear model to successfully forecast precipitation in the Japanese region [5]. Similarly, Liu Yong and colleagues utilized a three-layer BP neural network, integrating principal component analysis with precipitation and runoff data, to predict the autumn flood season with high stability and satisfactory accuracy [6]. Despite the BP neural network’s proven efficacy, its lengthy training time and the challenge of accurately approximating the error function due to discrepancies between input and target values remain significant obstacles [7,8,9].

A notable advancement is the application of the ConvLSTM model by Liang Zhenqing, Chen Sheng, and others for forecasting in the Guangzhou region [10]. ConvLSTM was proposed by Xingjian Shi, which synergizes the convolutional operations of CNNs with LSTM’s temporal data processing capabilities, effectively addressing the spatiotemporal dimensions of precipitation forecasting [11]. The results show that the correlation coefficient between the forecast results and the actual observed values remains above 0.6 within 1 h, and the false positive rate is less than 40%. The ConvLSTM model demonstrates a sophisticated handling of time series and spatial data, facilitating more accurate predictions of short-term precipitation events. In addition, the MOML algorithm developed by Zhang Pingwen’s team at Peking University demonstrates the potential of data mining and learning in intelligent and efficient forecasting, as demonstrated by its successful application in the Beijing 2022 Winter Olympics, where the algorithm improved the accuracy of model forecasting results for temperature, humidity, wind speed, and wind direction by more than 10% [12].

In this study, we combined the previous models and used deep learning methods for radar image extrapolation and realized a radar image extrapolation method based on ConvLSTM and SmaAT-UNet. First, we will introduce the SmaAT-UNet Model.

## 2. Introduction to SmaAT-UNet

**Figure 1:**
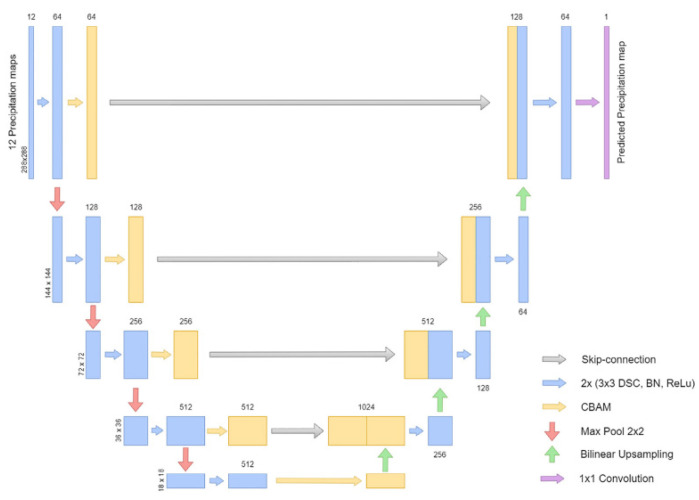
SmaAT-UNet network structure diagram.

SmaAT-UNet, an advanced model derived from the foundational UNet architecture, incorporates CBAM (Convolutional Block Attention Module) to enhance its decoder [13,14]. This module enables adaptive adjustments of feature map weights, ensuring tailored processing for specific tasks. Additionally, SmaAT-UNet innovates by replacing traditional convolution methods with depthwise separable convolution, significantly enhancing model efficiency. The structural blueprint of SmaAT-UNet is depicted in Figure 1.

CBAM combines a channel attention mechanism and spatial attention mechanism, is designed for visual applications such as image classification and object detection [15]. It addresses the challenge of uneven feature significance across various channels and spatial regions in convolutional neural networks. Through CBAM, the model emphasizes crucial channels and spatial locations by dynamically weighting feature information, thereby refining the focus and effectiveness of the network.

In detail, CBAM’s channel attention mechanism evaluates and assigns weights to each channel’s features using global average pooling and fully connected layers, producing a set of optimized channel features. Concurrently, the spatial attention mechanism assigns weights to specific spatial locations through convolutional and fully connected layers, crafting a targeted spatial feature set.

For the channel attention module, input features F are derived from both MaxPool and AvgPool operations, symbolizing the highest and average aggregated features, respectively. These features are then processed through a shared, fully connected layer, resulting in combined and weighted feature vectors, as defined by Equation (1):

(1) $$\begin{array}{ll} M_{c}(F) & =\sigma (MLP(AvgPool(F))+MLP(MaxPool(F)))\\ & =\sigma (W_{1}(W_{0}(F_{avg}^{c}))+W_{1}(W_{0}(F_{max}^{c}))) \end{array}$$

where **F**—Input feature, σ—Activation function and $W_{1}$, W0—Weight value.

The spatial attention module utilizes the feature vector from the channel attention module as its input. Post-processing through max-pooling and average-pooling, the results are merged and further refined by standard convolution, culminating in the generation of spatial attention weights. The procedural formula for spatial attention is outlined in Equation (2).

(2) $$\begin{array}{c} M_{s}(F)=\sigma (f^{7\times 7}([AvgPool(F);MaxPool(F)]))\\ =\sigma (f^{7\times 7}([F_{avg}^{s};F_{max}^{s}])) \end{array}$$

The CBAM model’s capability to adaptively discern and emphasize essential features across channels and spatial domains significantly bolsters SmaAT-UNet’s performance. Moreover, its low computational overhead allows for seamless integration into existing network frameworks.

SmaAT-UNet’s transition to depthwise separable convolution, comprising depthwise and pointwise convolutions, marks a departure from conventional convolution techniques. This modification enables individualized feature processing for each input channel and a subsequent amalgamation of these features, substantially reducing the model’s parameter count and computational demand. This strategic replacement underscores SmaAT-UNet’s streamlined and effective design, affirming its position as a lightweight yet powerful model for complex visual tasks.

## 3. Short-Term and Impending Precipitation Prediction Using Convolutional Long Short-Term Memory Network

### 3.1. Data Set Preparation

The efficacy of predictive models, particularly those extrapolating radar echo images for short-term precipitation forecasting, is fundamentally dependent on the quality of the dataset employed. Therefore, rigorous preprocessing of the dataset is indispensable to enhance the model’s accuracy and generalization capabilities. The original dataset may include erroneous or incomplete data, such as default values and outliers, which can compromise model performance. Through careful preprocessing, these inaccuracies can be mitigated to improve data quality significantly. Using data standardization and normalization and image filtering can improve the quality of data sets.

#### 3.1.1. Standardization and Normalization of Data

Normalization plays a crucial role in aligning data across different scales to a uniform standard, thus neutralizing scale-induced discrepancies in data analysis. This process is particularly critical in deep learning, where it ensures the equal importance of diverse features by mitigating issues arising from variance in feature value ranges, which could otherwise lead to inaccurate or slow model convergence. Moreover, normalization enhances model stability and interpretability.

The Z–R relationship, foundational to the empirical correlation between radar echo intensity and precipitation rate, facilitates the conversion of radar echo intensity to a standard precipitation rate formula. This pivotal conversion process is encapsulated in Equation (3).

(3) $$Z=aR^{b}$$

where Z—Radar echo intensity (in dBZ), R—Rainfall rate (in mm/h), a and b—Radar constant, with a ≈ 200 and b between 1.5 and 2.

#### 3.1.2. Image Filtering

The presence of noise in digital images can deteriorate image quality, adversely affecting subsequent image processing and analysis tasks. Excessive or irrelevant image details might also obscure experimental outcomes. To counter these issues, median filtering is employed to suppress noise, thereby augmenting image quality, eliminating superfluous details, and enhancing contrast. This technique, which replaces the gray value of each pixel with the median of the gray values in its vicinity, effectively highlights crucial image details for easier interpretation [16].

#### 3.1.3. Radar Dataset

This study utilizes the HKO-7 radar dataset, generously provided and licensed by the Hong Kong Observatory. This dataset has been subjected to thorough data cleaning and quality assurance measures, ensuring the reliability and accuracy of the data for forecasting purposes. To access, see “https://github.com/sxjscience/HKO-7 (accessed on 15 May 2024)”.

**Figure 2:**
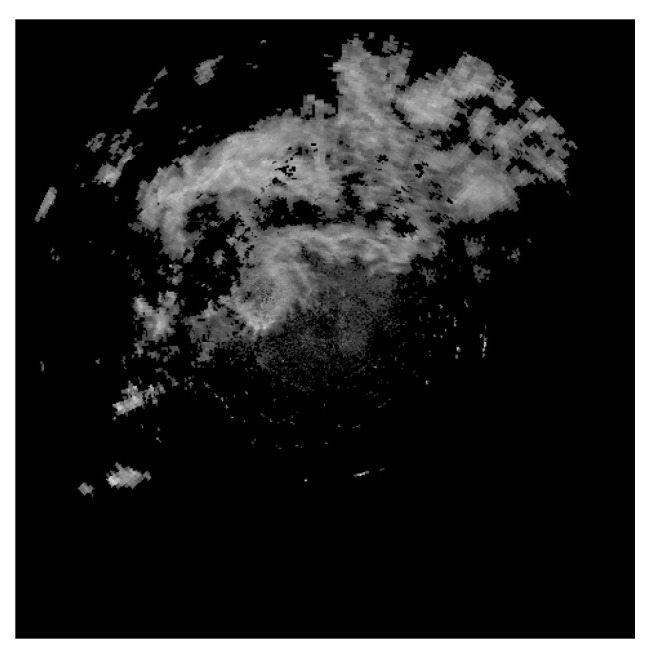
Radar gray echo data diagram.

The original radar echo images in this dataset are uniformly sized at 480 × 480 pixels. Considering the substantial volume and dimensions of the image data, which could prolong training durations, images are resized to 64 × 64 pixels. The 480 × 480 pixel radar echo images are illustrated in Figure 2.

Spanning from 2009 to 2015, the HKO-7 dataset encompasses radar echo data for the Hong Kong Special Administrative Region captured at 6-min intervals—equating to 240 data frames daily. The training model inputs 5 frames (30 min) to forecast the subsequent 20 frames (120 min). Due to computational constraints and the fact that rainfall in Hong Kong is concentrated from April to September, we selected two of these months: April and May [17]. A subset of data from April and May 2014, encompassing 61 days and 14,640 image frames, was randomly selected for model training.

### 3.2. Experimental Process

L1 regularization is the sum of the absolute values of each element in the weight vector w, and L2 regularization is the square root of the sum of squares in the weight vector. These two methods are to solve the overfitting problem, that is, to reduce the size or number of model parameters to alleviate the overfitting problem [18]. To mitigate the potential ambiguities associated with L1 and L2 regularization techniques, this study employs the Log–Cosh loss function. Known fully as “Logarithmic Hyperbolic Cosine Loss”, this function serves as a smooth alternative that effectively penalizes extreme values, thereby offering enhanced robustness compared to the Mean Squared Error (MSE) loss function. The mathematical representation of the Log–Cosh loss function is provided in Equation (4).

(4) $$L(y,y^{P})=\sum_{i=1}^{n}log(cosh(y_{i}^{P}-y_{i}))$$

where y denotes the actual values, y^p^ signifies the predicted values, and n represents the total number of samples. The parameter training process involves the dynamic adjustment of model parameters through the use of iterative algorithms such as the backpropagation (BP) algorithm. To circumvent overfitting, an early stopping mechanism is employed; this involves monitoring the validation dataset’s accuracy at the conclusion of each training iteration. Training ceases when there is no observed improvement in accuracy for n consecutive iterations, with n typically set at 10. Accordingly, for this experiment, the training configuration was established with 10,000 epochs, a batch size of 2, and the early stopping parameter n also set at 10.

### 3.3. Meteorological Evaluation Standard

The threshold to distinguish positive and negative samples, set at default to 0.1. We use a meteorological default grid point ≥ 0.1 to determine the presence of precipitation.

In the field of meteorology, it is not possible to simply use accuracy to describe the quality of a model. Instead, we mainly use the idea of “two-class classification” to evaluate it, comparing the prediction results with the actual observed results. We divide both the observed values and the predicted values into two categories: precipitation and no precipitation. If the pixel value of a pixel point in the radar image extrapolation result is greater than the observed echo intensity threshold, then we binarize it to 1, otherwise we binarize it to 0 [19].

The purpose of this is to facilitate our analysis and processing of the prediction results in order to better evaluate the accuracy and reliability of the prediction. False alarms are when the predicted value has precipitation, but the true observed value has no precipitation. Misses are when the true observed value has precipitation, but the predicted value does not have precipitation. Hits are when both the observed and predicted values have precipitation, and are determined to have precipitation. The classification is shown in Table 1.

POD represents the ratio of the predicted precipitation area to the total observed precipitation area, as shown in Equation (5).

(5) POD=Hits÷(Hits+Misses)

The False Alarm Rate (FAR) is the ratio of the predicted area of unobserved precipitation to the total area of observed precipitation, as shown in Equation (6).

(6) FAR=False alarms÷(Hits+False alarms)

The Critical Success Index (CSI) represents the proportion of correctly predicted precipitation areas to the total predicted precipitation area, as shown in Equation (7).

(7) CSI=Hits÷(Hits+Misses+False alarms)

### 3.4. Experimental Results

Evaluation of forecasting performance employs indicators such as Probability of Detection (POD), False Alarm Rate (FAR), and Critical Success Index (CSI).

**Figure 3:**
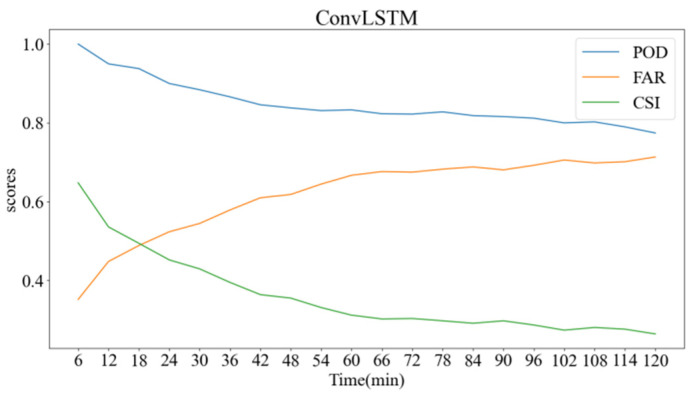
ConvLSTM predicts the change curve of three scores within two hours.

Our findings are systematically compiled in Table 2. Additionally, the ConvLSTM model’s predictive capabilities over a forthcoming two-hour interval, specifically regarding POD, FAR, and CSI changes, are graphically represented in Figure 3.

**Figure 4:**
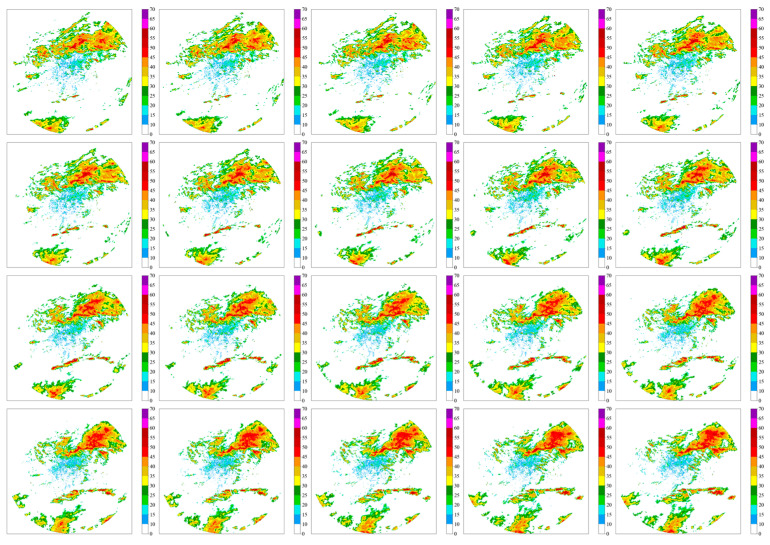
Observed radar image change diagram.

Figure 4 exhibits the observed radar imagery, providing a visual reference for the model’s predictive accuracy.

**Figure 5:**
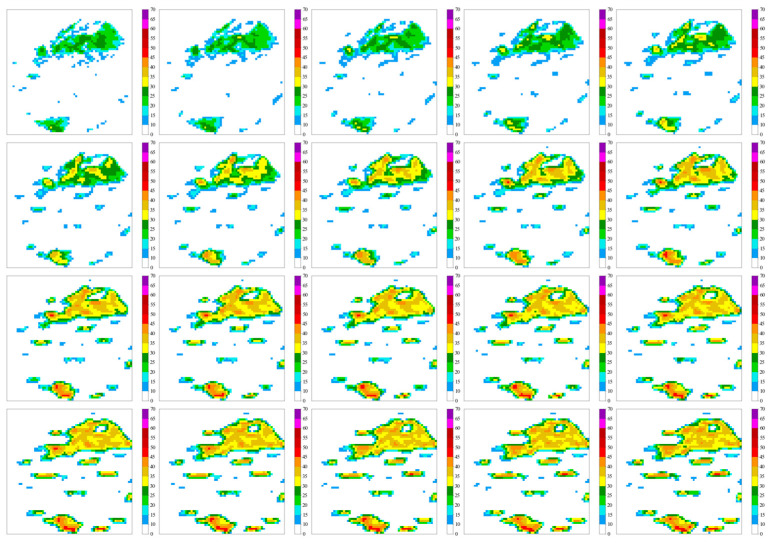
Predicted radar image changes using the ConvLSTM model.

Figure 5 below shows the radar image changes predicted by the ConvLSTM model.

## 4. Short-Term and Impending Precipitation Prediction Based on SmaAT-UNet

### 4.1. Backpropagation Optimization Algorithm—Adam Algorithm

The Adam algorithm stands as a pivotal optimization method for updating parameters within neural networks, distinguished by its adaptive learning rate mechanism. This algorithm, designed to dynamically adjust the learning rate based on the gradient variations of different parameters, significantly enhances both the efficiency and the generalizability of model training. By amalgamating the strengths of momentum gradient descent and the RMSProp algorithm, Adam achieves superior convergence rates and effects, as has been evidenced by studies [20,21].

Adam employs both first-order and second-order momentum in the parameter update process, facilitating an accelerated gradient descent approach. This dual momentum strategy allows Adam to not only swiftly converge towards optimal solutions but also to fine-tune the update trajectory and speed of model parameters with high precision [22].

Momentum Update Formula: The momentum update, integral to achieving accelerated descent, is formalized in Equation (8).

(8) ν(t)=μν(t−1)+(1−μ)g(t)

where ν(t) denotes the momentum of t times, μ refers to hyperparameters, and g(t) denotes the gradient value.

Adaptive Learning Rate Calculation: Adam calculates the adaptive learning rate by averaging the gradients and squared gradients, ensuring that the learning rate is optimally adjusted in response to parameter-specific gradient behaviors. The detailed calculation process is outlined in Equations (9)–(11).

(9) m(t)=β1m(t−1)+(1−β1)g(t)

(10) $$s(t)=\beta 2s(t-1)+(1-\beta 2)g^{2}(t)$$

(11) $$\alpha (t)=\frac{\eta \mu^{t}}{\sqrt{s(t)}+\varepsilon }$$

where $m\left(t\right),s\left(t\right),and\;\alpha (t)$ represent the mean average gradient moving average, square gradient moving average, and adaptive learning rate values, respectively; β1 and β2 are control gradient hyperparameters; μ and η are the momentum hyperparameters and initial learning rate, respectively; g(t) is the current gradient value of the batch; and ε is the offset term.

The algorithm’s ability to adaptively modulate the learning rate across different parameter gradients contributes to enhanced training efficiency and model generalization. Moreover, the incorporation of both first-order and second-order momentum enables Adam to adeptly manage the directionality and velocity of parameter updates, fostering improved model convergence dynamics.

### 4.2. Experimental Process

In the regression prediction of radar images, the distribution of radar reflectivity values is uneven, so the BMSE loss function is commonly used. In the regression prediction task, our goal is to predict continuous data images based on the input data, so we need to define a loss function to evaluate the gap between the predicted value and the true value. The BMSE loss function can achieve this [23]. The calculation formula of the BMSE loss function is shown in Equation (12).

(12) $$BMSE=\frac{1}{N}\sum_{n=1}^{N}\sum_{i=1}^{600}\sum_{j=1}^{600}\omega_{n,i,j}{\left(x_{n,i,j}-y_{n,i,j}\right)}^{2}$$

The Adam optimization algorithm, known for its adaptive learning rate, was utilized with specific parameters: learning rate (lr) of 0.001, betas of (0.9, 0.999), epsilon (eps) of 1 × 10^−8^, weight decay of 0, and AMSGrad set to False. An early stopping mechanism was implemented to prevent overfitting, with training parameters set to 10,000 epochs, a batch size of 4, and a patience parameter (n) of 10.

### 4.3. Experimental Result Comparison, Evaluation, and Analysis

Utilizing a consistent test dataset, we embarked on predicting precipitation for the forthcoming two hours through distinct methodologies. The acquired performance metrics of the models are systematically organized in the ensuing Table 3.

**Figure 6:**
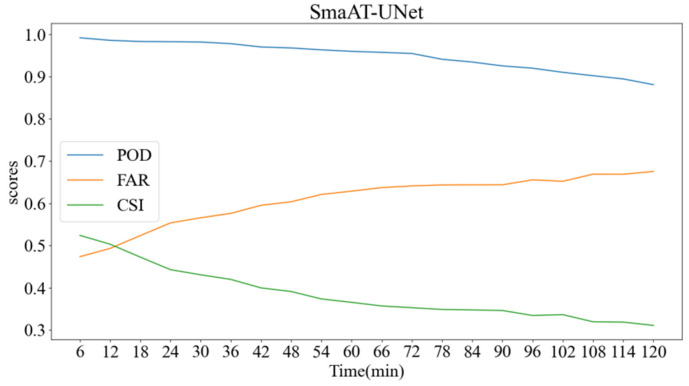
Three scoring curves of SmaAT-UNet predictions within two hours.

Furthermore, Figure 6 delineates the prediction dynamics of Probability of Detection (POD), False Alarm Rate (FAR), and Critical Success Index (CSI) over the specified period.

**Figure 7:**
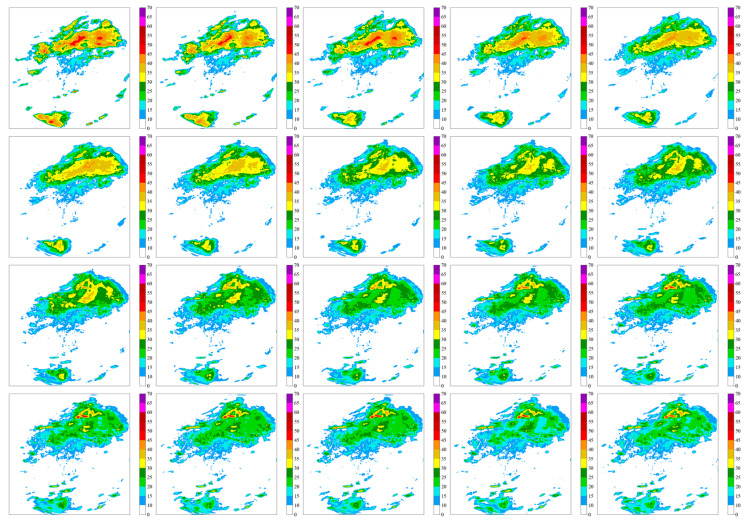
SmaAT-UNet predicted radar change map.

Figure 7 illustrates the variations in radar imagery as forecasted by the SmaAT-UNet model.

**Figure 8:**
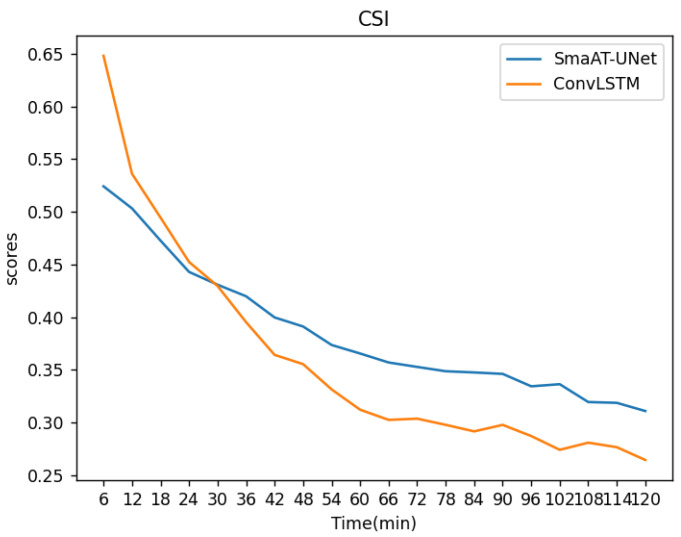
CSI change curves of the two methods.

An examination of the radar echoes, as depicted in Figure 8, reveals that within each six-minute increment, a total of 20 images span the two-hour forecast interval. A notable trend is the gradual decline in CSI scores over time, signifying a diminishing prediction accuracy with the extension of the forecast period. This trend underscores an increasing discrepancy between observed values and those predicted by the models. In the comparative analysis, the ConvLSTM model exhibited superior performance to SmaAT-UNet in the initial 30 min based on CSI metrics. Conversely, from the 30-minute mark to the 120-minute endpoint, SmaAT-UNet outperformed ConvLSTM. The average CSI metrics for both models, predicting the subsequent 20 frames, were approximately 0.38 and 0.36, respectively, with the CSI change curve indicating greater stability in the SmaAT-UNet model.

**Figure 9:**
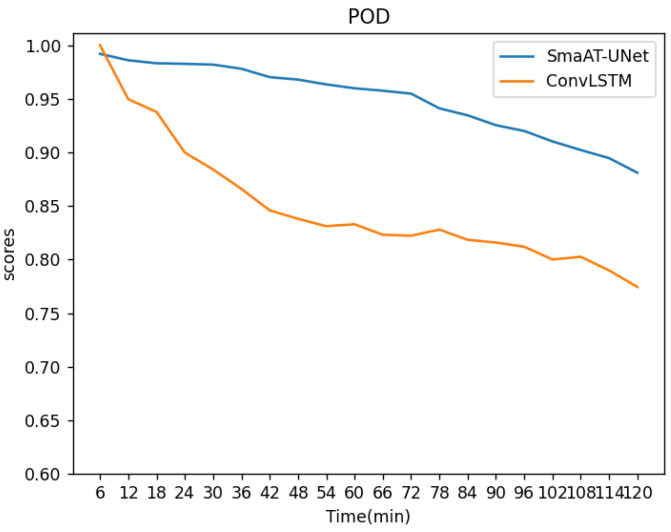
POD change curves of the two methods.

The evaluation of POD, a metric representing the hit rate, is visualized in Figure 9.

**Figure 10:**
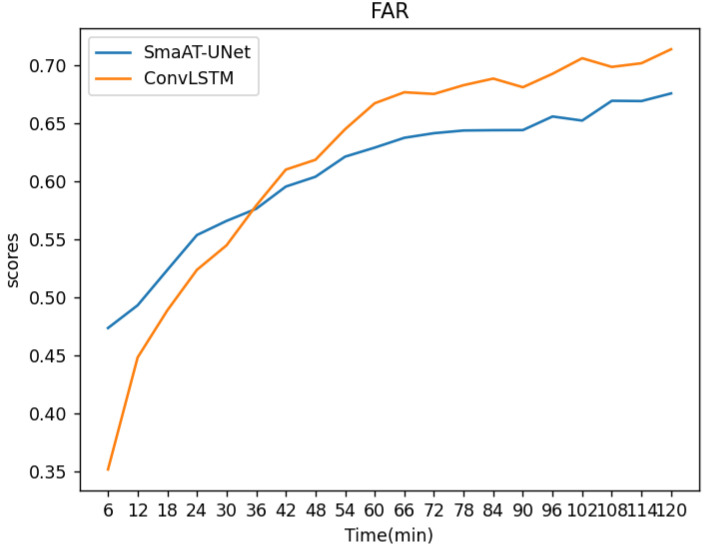
FAR change curves of the two methods.

This analysis indicates a declining hit rate for both models as time progresses, with SmaAT-UNet consistently outpacing ConvLSTM. Specifically, the average POD index for the first 20 frames was 0.85 for ConvLSTM and 0.95 for SmaAT-UNet. The FAR metrics, assessing the False Alarm Rate of radar echo extrapolation images predicted by the models, are detailed in Figure 10.

Initial observations up to the 36-minute mark suggest a higher false alarm rate for SmaAT-UNet compared to ConvLSTM, implying inferior performance. However, subsequent predictions show an inversion in this trend, with ConvLSTM exhibiting a higher false alarm rate than SmaAT-UNet. The average FAR indices for the first 20 frames were 0.62 for ConvLSTM and 0.61 for SmaAT-UNet, respectively.

## 5. Conclusions

In contrast to traditional radar image extrapolation methods that rely on physical models demanding extensive historical data, deep learning-based algorithms offer a robust alternative. These advanced algorithms require significantly less data for training, yet they yield superior predictive outcomes, demonstrating enhanced applicability in complex radar scenarios.

The ConvLSTM model merges the capabilities of Convolutional Neural Networks (CNNs) and Long Short-Term Memory (LSTM) networks to overcome LSTM’s limitations in processing long-term dependencies. While LSTM excels in handling time series data, CNNs adeptly manage spatial information. The SmaAT-UNet model further advances this approach by integrating the Convolutional Block Attention Module (CBAM) with UNet and adopting depthwise separable convolution, substantially reducing the model’s parameter count while maintaining efficacy for regression-based prediction tasks.

In summary, both the SmaAT-UNet and ConvLSTM models present promising solutions for short-term precipitation forecasting, with SmaAT-UNet exhibiting slight advantages in performance and stability. These findings advocate for the continued exploration and integration of deep learning methodologies in meteorological forecasting, paving the way for more accurate and reliable prediction models.

Future Directions:

- Exploration of Neural Network Architectures: Given the study’s scope constraints, only two deep learning models were examined. Future research should extend to comparing various neural network architectures known for efficacy in short-term precipitation forecasting. While the model parameters were optimized for the HKO-7 dataset, further refinement is recommended.
- Integration of Multi-Source Data: The dataset’s quality is pivotal to a model’s generalization capabilities. Deep learning models, typically data-hungry, face limitations due to the geographical and temporal constraints in meteorological data collection. Future endeavors could explore the incorporation of multi-source data, such as satellite and radar data, to enhance forecasting accuracy.
- Addressing ConvLSTM’s Limitations: Despite its advanced memory capabilities, ConvLSTM’s complex structure and substantial training demands limit its efficiency, particularly in managing long-term dependencies and processing radar image rotations and diffusions. The model’s sensitivity to input data variations also warrants attention. Future work should focus on addressing these challenges to improve model robustness and performance.

## Figures and Tables

**Table 1:** Comparison of Rainfall Classification.

	No Precipitation Forecasted	Precipitation Forecasted
No precipitation observed	Correct negatives	False alarms
Precipitation observed	Misses	Hits

**Table 2:** The average rating of the future 20 frames predicted by the ConvLSTM model.

	Evaluation Indicators	POD	FAR	CSI
Model				
ConvLSTM		0.85	0.62	0.36

**Table 3:** Average scores of ConvLSTM and SmaAT-UNet models in predicting the next 20 frames.

	Evaluation Indicators	POD	FAR	CSI
Model				
ConvLSTM		0.85	0.62	0.36
SmaAT-UNet		0.95	0.61	0.38

## Data Availability

The original contributions presented in the study are included in the article, further inquiries can be directed to the corresponding author.
